# Estimated Trans-Lamina Cribrosa Pressure Differences in Low-Teen and High-Teen Intraocular Pressure Normal Tension Glaucoma: The Korean National Health and Nutrition Examination Survey

**DOI:** 10.1371/journal.pone.0148412

**Published:** 2016-02-03

**Authors:** Si Hyung Lee, Seung Woo Kwak, Eun Min Kang, Gyu Ah Kim, Sang Yeop Lee, Hyoung Won Bae, Gong Je Seong, Chan Yun Kim

**Affiliations:** 1 Institute of Vision Research, Department of Ophthalmology, Severance Hospital, Yonsei University, College of Medicine, Seoul, Korea; 2 Department of Statistics, University of Georgia, Athens, Georgia; Duke University, UNITED STATES

## Abstract

**Background:**

To investigate the association between estimated trans-lamina cribrosa pressure difference (TLCPD) and prevalence of normal tension glaucoma (NTG) with low-teen and high-teen intraocular pressure (IOP) using a population-based study design.

**Methods:**

A total of 12,743 adults (≥ 40 years of age) who participated in the Korean National Health and Nutrition Examination Survey (KNHANES) from 2009 to 2012 were included. Using a previously developed formula, cerebrospinal fluid pressure (CSFP) in mmHg was estimated as 0.55 × body mass index (kg/m^2^) + 0.16 × diastolic blood pressure (mmHg)—0.18 × age (years)—1.91. TLCPD was calculated as IOP–CSFP. The NTG subjects were divided into two groups according to IOP level: low-teen NTG (IOP ≤ 15 mmHg) and high-teen NTG (15 mmHg < IOP ≤ 21 mmHg) groups. The association between TLCPD and the prevalence of NTG was assessed in the low- and high-teen IOP groups.

**Results:**

In the normal population (n = 12,069), the weighted mean estimated CSFP was 11.69 ± 0.04 mmHg and the weighted mean TLCPD 2.31 ± 0.06 mmHg. Significantly higher TLCPD (p < 0.001; 6.48 ± 0.27 mmHg) was found in the high-teen NTG compared with the normal group. On the other hand, there was no significant difference in TLCPD between normal and low-teen NTG subjects (p = 0.395; 2.31 ± 0.06 vs. 2.11 ± 0.24 mmHg). Multivariate logistic regression analysis revealed that TLCPD was significantly associated with the prevalence of NTG in the high-teen IOP group (p = 0.006; OR: 1.09; 95% CI: 1.02, 1.15), but not the low-teen IOP group (p = 0.636). Instead, the presence of hypertension was significantly associated with the prevalence of NTG in the low-teen IOP group (p < 0.001; OR: 1.65; 95% CI: 1.26, 2.16).

**Conclusions:**

TLCPD was significantly associated with the prevalence of NTG in high-teen IOP subjects, but not low-teen IOP subjects, in whom hypertension may be more closely associated. This study suggests that the underlying mechanisms may differ between low-teen and high-teen NTG patients.

## Introduction

Open-angle glaucoma (OAG) is a major chronic optic neuropathy characterized by progressive retinal ganglion cell loss and optic nerve head structural changes. It is the leading cause of irreversible blindness worldwide [1], and the associated economic impact is likely to increase in the future because of the aging global population [2]. However, the mechanisms underlying the development of glaucomatous optic neuropathy remain to be elucidated.

Recent experimental and clinical studies have reported that cerebrospinal fluid pressure (CSFP) may play an important role in the pathogenesis of glaucomatous optic neuropathy [3–10]. Moreover, several studies have demonstrated that glaucomatous optic nerve damage may develop in subjects with normal IOP due to abnormally low CSFP [6, 7]. Since optic nerve head and lamina cribrosa are located at the junction between the relatively high-pressure intraocular space and low-pressure subarachnoid space [11], pressure imbalance between these two regions, known as the trans-lamina cribrosa pressure difference (TLCPD), may be an important cause of glaucomatous optic nerve damage, especially in subjects with normal-tension glaucoma (NTG).

In Asian populations, more attention is paid to NTG because of its high prevalence in Asian countries [12–16]. Although the development and progression of NTG is affected by IOP, NTG is considered to be affected by non-IOP factors as well, such as vascular factors. The exact pathophysiology of NTG remains unclear, but it is generally known to be multifactorial. To reveal the specific underlying pathologic mechanisms, several studies further differentiated NTG into two categories with different clinical characteristics: low-teen NTG (NTG with IOP ≤15 mmHg) and high-teen NTG (NTG with 15 mmHg < IOP ≤ 21 mmHg) [17–21], providing indirect evidence that the mechanism underlying glaucomatous optic neuropathy in NTG may differ between subjects with low- and high-teen IOP.

In light of the above, we investigated whether TLCPD, as compared with IOP, is more strongly associated with low-teen IOP or high-teen IOP NTG patients using a population-based study design. Additionally, we further sought possible associations between systemic vascular diseases, which are major risk factors for NTG, and the prevalence of glaucoma in the two IOP groups. Since measurement of CSFP requires an invasive procedure, we estimated CSFP and calculated TLCPD using a formula published previously by Xie *et al*., which is based on three parameters: age, body mass index (BMI), and diastolic blood pressure [22].

## Materials and Methods

### Study population

The study was based on the Korean National Health and Nutrition Examination Survey (KNHANES), which is a nationwide, population-based and cross-sectional health examination and survey conducted by the Korea Centers for Disease Control and Prevention with approval from its Institutional Review Board [23]. The KNHANES uses a multistage, stratified, probability-clustered sampling method. The survey design can produce estimated health statistics representative of the entire South Korean population. The survey adhered to the principles outlined in the Declaration of Helsinki for research involving human subjects, and all participants provided written informed consent. The Institutional Review Board (IRB)/Ethics Committee of Yonsei University Health System approval was obtained.

The KNHANES was performed in 1998 (KNHANES I), 2001 (KNHANES II), 2005 (KNHANES III), 2007–2009 (KNHANES IV), and 2010–2012 (KNHANES V). The survey consists of four main components: a health interview, a health behavior survey, a health examination, and a nutrition survey. The interview includes questions regarding demographic, socioeconomic, health, and nutritional information. Examinations include vital signs, physiologic measurements, and basic laboratory tests. Ophthalmologic interview and examination data were available from 2008.

This analysis included 33,136 individuals from the fourth (KNHANES IV-3, 2009) and fifth (KHANES V-1-3, 2010, 2011, 2012) KNHANES, together with ophthalmologic examination data. Among these participants, we restricted the study population to those who met the following inclusion criteria: 40 years of age or older; no previous diagnosis with glaucoma; no anti-glaucoma medication usage; and no history of refractive, retinal or glaucoma surgery.

### Components of the ophthalmic survey

All participants underwent ophthalmologic interviews, visual acuity measurements, IOP measurements, auto-refraction, slit-lamp examination, and fundus photography. The spherical equivalent (SE) refractive error was calculated as sphere + 1/2 cylinder. Bilateral IOP was measured once, from right to left, by a trained ophthalmologist using a Goldmann applanation tonometer (Haag-Streit model BQ-900; Haag-Streit, Inc., Bern, Switzerland) during slit-lamp examination.

Digital fundus images were obtained using a digital non-mydriatic retinal camera (TRC-NW6S; Topcon, Inc., Tokyo, Japan) and a Nikon D-80 digital camera (Nikon, Inc., Tokyo, Japan). Based on the fundus images, vertical cup-to-disc ratios (VCDRs) and horizontal cup-to-disc ratios were measured. Participants with any signs of diabetic retinopathy or age-related macular degeneration were excluded from the study.

A visual field test with frequency double technology (FDT) was performed in participants who met the following glaucoma suspicion criteria: (1) IOP ≥ 22 mmHg, (2) horizontal or VCDR ≥ 0.5, (3) violation of the ISNT rule, i.e., that normal eyes show a characteristic configuration of neuroretinal rim thickness in the following order: inferior > superior > nasal > temporal, (4) presence of optic disc hemorrhage, or (5) presence of retinal nerve fiber layer defects. Participants were considered to have open-angle glaucoma based on the modified International Society of Geographical and Epidemiological Ophthalmology (ISGEO) criteria for the Korean population [14, 24]. The specific diagnostic criteria were as follows. Category 1 requires both (1) a reliable visual field defect consistent with glaucoma (fixation error and false positive error ≤ 1 and the presence of at least one location of reduced sensitivity) (2) and glaucomatous optic disc (neuroretinal rim loss with VCDR or horizontal cup-disc ratio ≥ 0.6 or the presence of optic disc hemorrhage or the presence of retinal nerve fiber layer defect or asymmetry of VCDR ≥ 0.2). Category 2, if a visual field test is not available or not reliable (fixation error or false positive error ≥ 2), requires (1) VCDR ≥ 0.9 or (2) asymmetry of VCDR ≥ 0.3 or (3) the presence of retinal nerve fiber layer defect and violation of the ISNT rule. Subjects with a shallow anterior chamber (peripheral anterior chamber depth ≤ 1/4) were excluded. From the entre glaucoma group, those with NTG (IOP ≤ 21mmHg) were selected and further differentiated into low-teen NTG (IOP < 15 mmHg) and high-teen NTG (IOP ≥ 15 mmHg) subjects.

### Estimation of cerebrospinal fluid pressure

For the estimation of CSFP, we used a formula published previously by Xie *et al*. in a pilot study [22]. They formed an algorithm for determining CSFP based on three parameters, diastolic BP, BMI, and age (estimated CSFP [mmHg] = 0.44 × BMI [kg/m^2^] + 0.16 × diastolic blood pressure [mmHg]—0.18 × age [years]—1.91), and applied the formula to groups of subjects by comparing the estimated CSFP with the direct CSFP measurements. Using the calculated CSFP, TLCPD was calculated as IOP -CSFP.

### Other traits

Demographic variables included age, gender, urban or rural region of residence, income and education level. Income status was quantified by quartile, and education level was categorized as elementary school or less, middle school graduate, high school graduate, or college graduate and beyond.

Other variables included in this study were anthropometric measurements, such as BMI; health-related behaviors, such as smoking, and exercise; and medical comorbidities, such as hypertension, and diabetes mellitus. Fasting glucose were assessed from blood samples, and systolic and diastolic blood pressure (BP) were measured using a standard mercury sphygmomanometer (Baumanometer, WA Baum Co., New York, USA). The average of two systolic and diastolic BP, obtained at an interval of 5 minutes, was used for analyses. Subjects were defined as having hypertension if they had a history of taking antihypertensive medication, or when the measured blood pressure (BP) was ≥ 140/90 mmHg. Subjects with hypertension were further categorized into two subgroups: 1) participants with actual hypertension (systolic/diastolic BP ≥ 140/90 in the absence of antihypertensive medication), and 2) participants taking antihypertensive medication. Participants who had been diagnosed with diabetes by physicians or had a fasting glucose level ≥ 126 mg/dL were defined as having diabetes mellitus. In addition, refractive status was classified into following four categories: 1) emmetropia (−0.99—+0.99 diopter [D]); 2) mild myopia (−1.00–−2.99D); 3) moderate myopia (−3.00—−5.99 D); 4) high myopia (≤−6.00 D); and 5) hyperopia (≥+1.00 D) [25].

### Statistical analysis

Complex sample analysis was performed using the SAS statistical software (version 9.3; SAS Institute, Inc., Cary, NC, USA) to provide nationally representative prevalence estimates. The demographic characteristics of the participants according to the presence and types of glaucoma were compared using the Rao-Scott χ^2^ test for categorical variables and Wald tests for continuous variables. The weighted mean values of IOP, CSFP, and TLCPD were compared between the normal control and NTG groups as well as among the normal control, low-teen NTG and high-teen NTG groups. Multivariate logistic regression analyses were further conducted to assess the association between the prevalence of glaucoma and IOP or TLCPD in the two IOP groups. To this end, the normal control group was also stratified into two groups based on the IOP level. Three different adjusted models for analysis were developed. Model 1 represented crude odds ratio (OR), and Model 2 was adjusted for age and sex. In model 3, further adjustments were made for other potential covariates, including hypertension, smoking status, refractive status, and education level. ORs and 95% confidence intervals (CI) were presented. All variables for logistic regression analysis were tested for multicollinearity, and only variables with a variance inflation factor ≤ 5 were included. In all analyses, p values were two-tailed and those < 0.05 were considered to indicate statistical significance.

## Results

**Fig 1:**
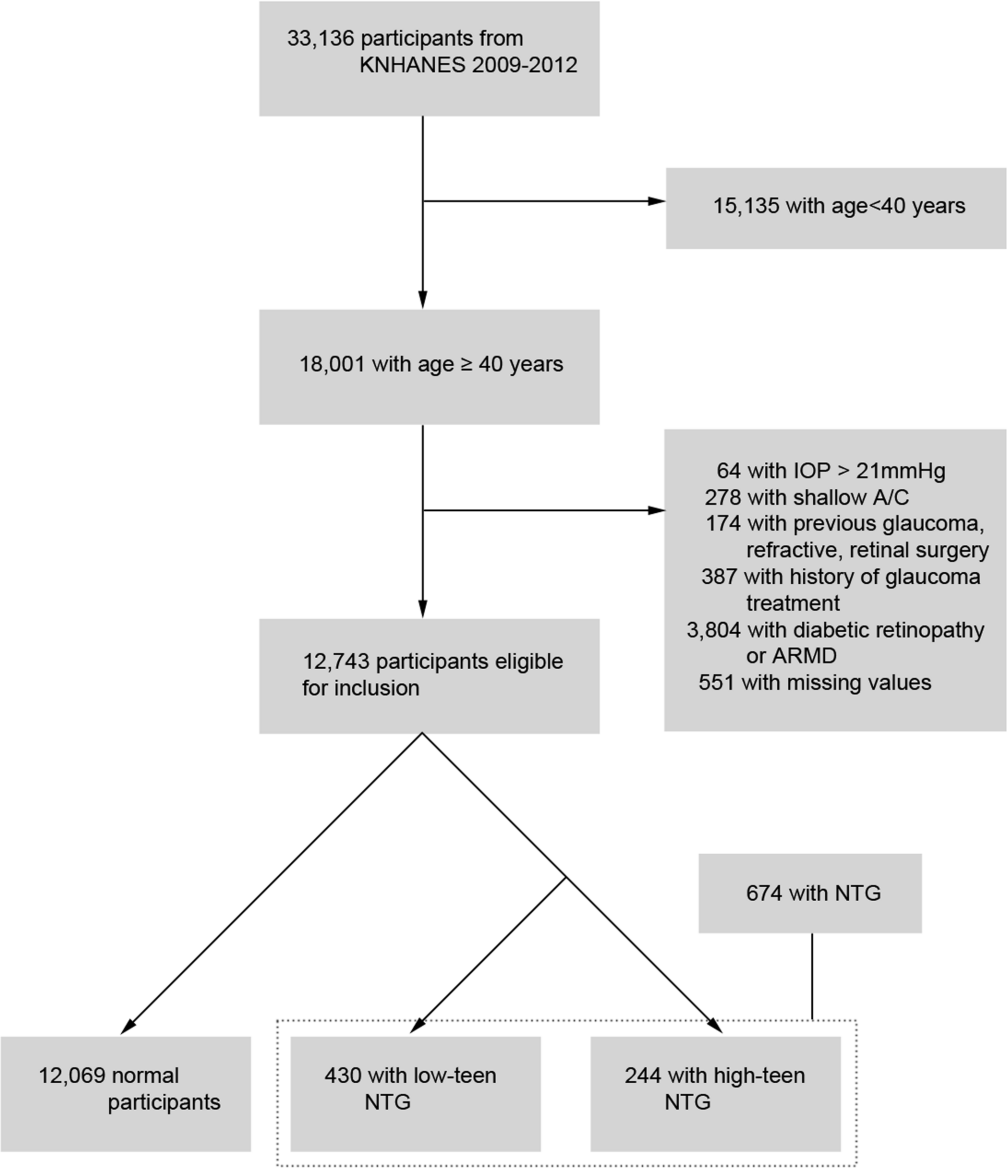
Flow diagram of inclusion and exclusion of the study participants. Following application of the exclusion criteria, a final total of 12,743 normal participants and 674 participants with normal pressure open-angle glaucoma were enrolled in this study. KNHANES, Korean National Health and Nutrition Examination Survey; BMI, body mass index; IOP, intraocular pressure; A/C, anterior chamber; NTG, normal tension glaucoma; OAG, open-angle glaucoma; ARMD, age-related macular degeneration.

Among 33,136 participants who underwent ophthalmologic examinations, 18,001 subjects ≥ 40 years of age were included in the analyses. Participants with the following conditions were further excluded: IOP > 21 (n = 64); history of glaucoma or refractive or retinal surgery (n = 174); history of glaucoma medical treatment (n = 387), shallow peripheral angle (n = 278); any findings of diabetic retinopathy or age-related macular degeneration (n = 3,804); and missing values (n = 551). Thus 12,743 participants were eligible for inclusion. Participants were then further grouped into a non-glaucomatous group (n = 12,069) and NTG group (n = 674) (Fig 1).

The demographic characteristics and weighted mean values of the systemic and ocular parameters are shown in Table 1. The NTG group was again divided into two groups: the low-teen NTG (n = 430) and high-teen NTG (n = 244) groups. Participants with NTG, both low-teen and high-teen IOP, were significantly older (p < 0.001), male predominant (p = 0.001), less educated (p < 0.001), had higher prevalence of hypertension (p < 0.001), and had more myopic refractive error (p < 0.001) compared with normal participants.

**Table 1:** Demographics and general health characteristics of the normal and glaucomatous populations.

Parameter	Normal (n = 12,069)	NTG (n = 674)			*P*^†^
			Low-teen NTG (n = 430)	High-teen NTG (n = 244)	
Demographics					
Age, years					< .001*
40–49	42.9 (0.8)	23.7 (2.3)*	18.8 (2.6)*	30.6 (4.1)*	
50–59	31.8 (0.6)	32.7 (2.2)	32.1 (2.8)	33.6 (3.8)	
60–69	15.3 (0.4)	21.4 (1.6)	23.9 (2.2)	18.0 (2.7)	
≥70	10.0 (0.3)	22.1 (1.7)	25.3 (2.3)	17.7 (2.7)	
Female, %	51.9 (0.5)	44.1 (2.4)*	45.3 (3.8)*	34.3 (3.7)*	0.001*
Area of residence, %					0.463
Urban region	67.0 (1.1)	68.7 (2.4)	66.8 (2.9)	71.3 (3.8)	
Rural region	33.0 (1.1)	31.3 (2.4)	33.2 (2.9)	28.7 (3.8)	
Education, %					< .001*
≤Elementary school	27.0 (0.7)	38.9 (2.4)*	43.7 (3.0)*	32.2 (3.8)*	
Middle school graduate	15.8 (0.4)	14.6 (1.7)	13.4 (2.0)	16.3 (2.9)	
High school graduate	34.9 (0.7)	26.2 (2.2)	24.2 (2.7)	28.9 (3.7)	
≥College graduate	22.2 (0.7)	20.4 (2.2)	18.7 (2.5)	22.6 (3.7)	
Income, %					0.193
Quartile 1	25.5 (0.6)	29.3 (2.2)	26.9 (2.6)	32.7 (3.7)	
Quartile 2	26.5 (0.6)	22.8 (1.9)	23.0 (2.4)	22.5 (3.0)	
Quartile 3	24.4 (0.5)	25.1 (2.1)	25.0 (2.7)	25.2 (3.4)	
Quartile 4	23.6 (0.7)	22.8 (2.1)	25.1 (2.5)	19.6 (3.4)	
Smoking status, %					0.024*
Current	22.8 (0.5)	24.0 (2.4)*	22.4 (2.8)	26.2 (4.0)*	
Former	22.4 (0.5)	26.7 (2.1)	23.5 (2.5)	31.2 (3.8)	
Never	54.8 (0.5)	49.3 (2.4)	54.1 (3.0)	42.6 (3.9)	
Exercise days per week, %					0.224
0	64.4 (1.1)	71.6 (2.3)	74.0 (2.7)	68.3 (3.9)	
1	10.9 (0.7)	9.0 (1.6)	7.6 (1.5)	11.1 (3.2)	
2	8.3 (0.6)	7.3 (1.3)	6.8 (1.8)	8.0 (2.1)	
3	7.1 (0.6)	4.8 (0.9)	5.4 (1.3)	4.0 (1.3)	
≥4	9.4 (0.6)	7.2 (1.3)	6.2 (1.4)	8.6 (2.3)	
BMI, %					0.198
<25 kg/m^2^	63.7 (0.6)	66.8 (2.3)	65.2 (2.8)	69.0 (3.6)	
≥25 kg/m^2^	36.3 (0.6)	33.2 (2.3)	34.8 (2.8)	31.0 (3.6)	
Medical comorbidities, %					
Diabetes mellitus	10.0 (0.4)	11.7 (1.5)	12.0 (1.7)	11.4 (2.6)	0.231
Hypertension	22.5 (0.5)	37.1 (2.2)*	40.4 (2.9)*	32.5 (3.5)*	< .001*
Refractive status^‡^					< .001*
Emmetropia	58.3 (0.6)	49.8 (2.4)*	52.6 (3.0)*	45.8 (4.1)*	
Myopia					
Mild	18.8 (0.5)	16.0 (1.8)	14.9 (2.3)	17.5 (3.0)	
Moderate	6.2 (0.3)	11.2 (1.8)	7.3 (1.6)	16.8 (3.5)	
Severe	3.3 (0.2)	8.3 (1.5)	7.4 (1.8)	9.6 (3.5)	
Hyperopia	13.4 (0.4)	14.7 (1.5)	17.8 (2.0)	10.4 (2.0)	
Pressure parameters, mmHg					
Estimated CSFP	11.69 (0.04)	10.76 (0.16)*	10.45 (0.20)*	10.97 (0.30)*	< .001*
IOP	14.01 (0.05)	14.59 (0.16)*	12.56 (0.11)*	17.45 (0.16)*	< .001*
TLCPD	2.31 (0.06)	3.82 (0.21)*	2.11 (0.24)	6.48 (0.27)*	< .001*

NTG, normal tension glaucoma; IOP, intraocular pressure; BMI, body mass index; CSFP, cerebrospinal fluid pressure; TLCPD, trans-lamina cribrosa pressure difference. All means and frequency (%) are weighted estimates with standard errors.

*P<0.05 versus the normal control group.

^†^Rao-Scott χ^2^ test (for categorical variables) or Wald test (for continuous variables) was used.

^‡^ Emmetropia (-0.99 to 0.99 D in spherical equivalent), mild myopia (-1.00 to -2.99 D), moderate myopia (-3.00 to -5.99 D), severe myopia (<-6.00 D), and hyperopia (≥1.00 D).

**Fig 2:**
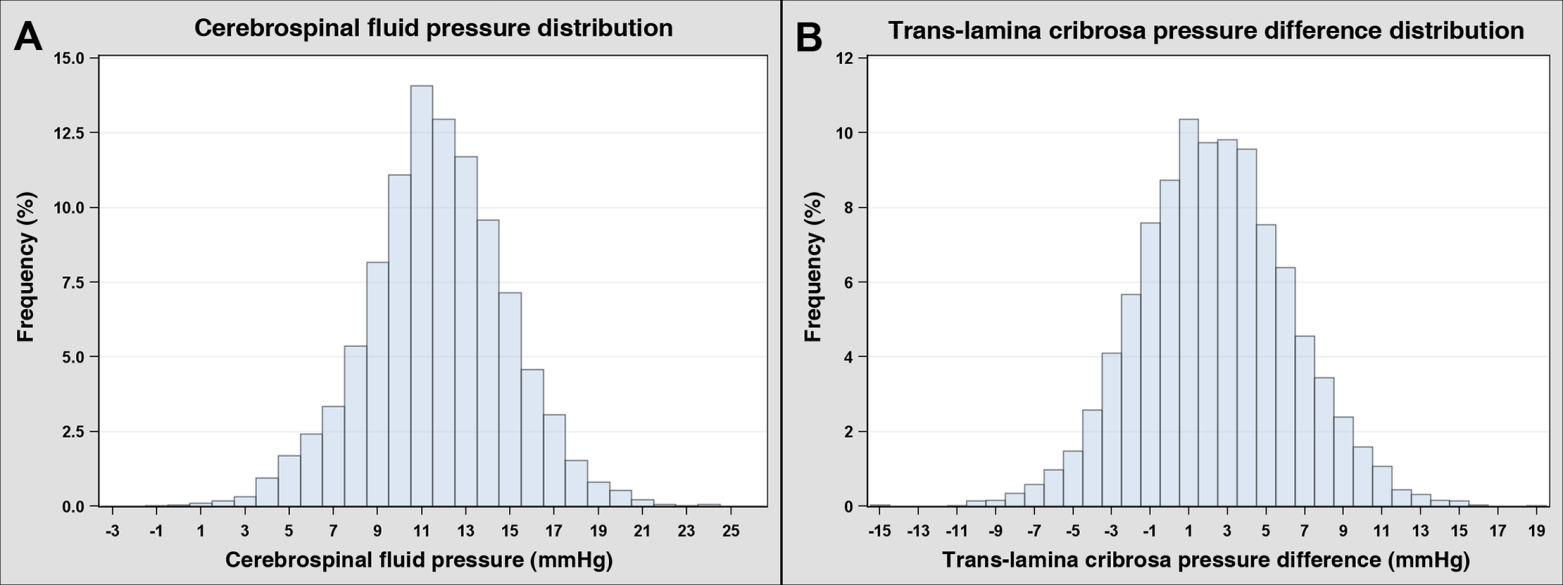
Distribution of the estimated cerebrospinal fluid pressure (A) and trans-lamina cribrosa pressure difference (B) in normal subjects of the Korean National Health and Nutrition Examination Survey. The weighted mean estimated cerebrospinal fluid pressure was 11.69 ± 0.04 mmHg, and the weighted mean trans-lamina cribrosa pressure difference was 2.31 ± 0.06 mmHg.

In the normal population, the weighted mean estimated CSFP value was 11.69 ± 0.04 mmHg (range: -1.80–23.91 mmHg), and the weighted mean TLCPD was 2.31 ± 0.06 mmHg (range: -14.61–19.11 mmHg) (Fig 2). Comparison of the three pressure parameters—IOP, estimated CSFP, and TLCPD—between the normal control and NTG groups revealed significant differences in all three (IOP: 14.01 ± 0.05 vs. 14.59 ± 0.16 mmHg, p < 0.001; estimated CSFP: 11.69 ± 0.04 vs. 10.76 ± 0.16 mmHg, p < 0.001; TLCPD: 2.31 ± 0.06 vs. 3.82 ± 0.21 mmHg, p < 0.001, respectively) (Table 1). There were significant differences in IOP between the normal and low-teen NTG groups (14.01 ± 0.05 vs. 12.56 ± 0.11 mmHg, respectively; p < 0.001), the normal and high-teen NTG groups (14.01 ± 0.05 vs. 17.45 ± 0.16 mmHg, respectively; p < 0.001), and the low-teen NTG and high-teen NTG groups (p < 0.001). When estimated CSFP was considered, the low-teen NTG and high-teen NTG groups showed significantly lower CSFP levels compared with normal subjects (10.45 ± 0.20 vs. 10.97 ± 0.30 vs. 11.69 ± 0.04 mmHg, respectively; p < 0.001), and a significant difference between the two NTG groups was also observed (p < 0.001). On the other hand, TLCPD in the low-teen NTG group did not differ significantly from that in the normal subjects (2.11 ± 0.24 mmHg versus 2.31 ± 0.06 mmHg; p = 0.395), while TLCPD in the high-teen NTG group (6.48 ± 0.27 mmHg) was significantly higher than that in the normal control and low-teen NTG groups (p < 0.001).

To investigate the association between TLCPD and the prevalence of NTG in the low-teen and high-teen IOP groups, a multivariate binary logistic regression analysis was conducted. Together with the potential confounding covariates, three adjusted models were tested. Both IOP and TLCPD were significantly associated with the NTG prevalence, with more statistically significance and slightly higher odds for TLCPD in fully adjusted Model 3 (p = 0.016; OR: 1.05; 95% CI: 1.02, 1.12; p < 0.001; OR: 1.06; 95% CI: 1.02, 1.09, respectively) (Table 2). In the high-teen IOP group, the logistic regression analysis in Model 3 demonstrated that only TLCPD was significantly associated with NTG (p = 0.006; OR: 1.09; 95% CI: 1.02, 1.15) (Table 2), while analysis for IOP did not reveal any statistically significance in fully adjusted model (p = 0.247; OR: 1.08; 95% CI: 0.95, 1.23). On the other hand, in the low-teen IOP group, adjusted analysis in both Models 2 and 3 did not show any significant association between NTG and IOP (p = 0.687 and 0.450, respectively) or between NTG and TLCPD (p = 0.375 and 0.636, respectively).

**Table 2:** Odds ratios for the presence of glaucoma according to intraocular pressure and trans-lamina cribrosa pressure difference.

	Intraocular pressure			Trans-lamina cribrosa pressure difference		
	OR	95% CI	*p*	OR	95% CI	*p*
NTG						
^†^Model 1	1.09	1.04, 1.13	< .001*	1.10	1.07, 1.13	< .001*
^‡^Model 2	1.07	1.03, 1.13	< .001*	1.05	1.02, 1.08	0.003*
^§^Model 3	1.05	1.02, 1.12	0.016*	1.06	1.02, 1.09	< .001*
Low-teen NTG						
^†^Model 1	0.97	0.91, 1.04	0.392	1.08	1.04, 1.12	< .001*
^‡^Model 2	0.99	0.92, 1.06	0.687	0.99	0.94, 1.03	0.375
^§^Model 3	0.98	0.91, 1.04	0.450	0.99	0.94, 1.04	0.636
High-teen NTG						
^†^Model 1	1.13	1.03, 1.25	0.013*	1.10	1.05, 1.15	< .001*
^‡^Model 2	1.09	1.00, 1.25	0.048*	1.09	1.02, 1.15	0.014*
^§^Model 3	1.08	0.95, 1.23	0.247	1.09	1.02, 1.15	0.006*

NTG, normal tension glaucoma.

*P < 0.05.

^†^Model 1: crude odd ratios.

^‡^Model 2: adjusted for age, and sex.

^§^Model 3: adjusted for age, sex, hypertension, smoking status, refractive status, and education level.

Systemic vascular diseases are also well-known risk factors for development of NTG [26–30]. Considering the insignificant association between the prevalence of NTG and IOP or TLCPD in the low-teen IOP group, we further investigated any possible association between the NTG prevalence and the presence of systemic vascular disease. Hypertension and diabetes mellitus (DM) were included as independent variables and the NTG prevalence as a dependent variable. In low-teen IOP group, hypertension was significantly associated with NTG in fully adjusted for age, sex, smoking status, refractive status, and education level (p < 0.001; OR: 1.65; 95% CI: 1.26, 2.16), whereas for DM it did not (p = 0.939; OR: 0.99; 95% CI: 0.69, 1.40) (Table 3). The high-teen IOP group, however, did not show any statistically significant association between NTG and the presence of systemic vascular diseases after adjustment for the confounding factors (Hypertension: p = 0.200, DM: p = 0.462).

**Table 3:** Odds ratios for the prevalence of glaucoma according to the presence of hypertension, and diabetes mellitus.

	Hypertension			Diabetes mellitus		
	OR	95% CI	*p*	OR	95% CI	*p*
NTG						
^†^Model 1	2.03	1.67, 2.46	< .001*	1.14	0.78, 1.65	0.501
^‡^Model 2	1.50	1.21, 1.83	< .001*	0.94	0.69, 1.26	0.657
^§^Model 3	1.50	1.22, 1.84	< .001*	0.94	0.70, 1.27	0.677
Low-teen NTG						
^†^Model 1	2.37	1.85, 3.03	< .001*	1.29	0.84, 1.97	0.242
^‡^Model 2	1.66	1.23, 2.10	< .001*	0.99	0.70, 1.41	0.973
^§^Model 3	1.65	1.26, 2.16	< .001*	0.99	0.69, 1.40	0.939
High-teen NTG						
^†^Model 1	1.59	1.15, 2.20	0.005*	0.90	0.46, 1.75	0.656
^‡^Model 2	1.26	0.89, 1.78	0.187	0.80	0.47, 1.35	0.405
^§^Model 3	1.25	0.89, 1.75	0.200	0.82	0.49, 1.38	0.462

NTG, normal tension glaucoma.

*P < 0.05.

^†^Model 1: crude odd ratios.

^‡^Model 2: adjusted for age, and sex.

^§^Model 3: adjusted for age, sex, smoking status, refractive status, and education level.

Next, we further carried out subgroup analyses to seek for specific association between NTG prevalence and hypertension. Participants with hypertension were categorized into two groups; subjects with actual hypertension and those who are taking antihypertensive medication. Fully adjusted model revealed taking antihypertensive medication was positively associated with NTG prevalence in low-teen IOP group (p = 0.001; OR: 1.56; 95% CI: 1.19, 2.05), while actual hypertension was not (p = 0.472; OR: 1.14; 95% CI: 0.79, 1.64) (Table 4). In high-teen IOP group, both actual hypertension and taking antihypertensive medication were not associated with NTG prevalence in fully adjusted model (p = 0.800 and p = 0.345, respectively).

**Table 4:** Association between glaucoma and actual hypertension or taking antihypertensive medication.

	Actual hypertension			Taking antihypertensive medication		
	OR	95% CI	*p*	OR	95% CI	*p*
NTG						
^†^Model 1	1.28	0.97, 1.69	0.078	1.97	1.62, 2.40	<0.001*
^‡^Model 2	1.05	0.79, 1.40	0.722	1.42	1.15, 1.75	0.001*
^§^Model 3	1.08	0.81, 1.45	0.589	1.42	1.15, 1.76	0.001*
Low-teen NTG						
^†^Model 1	1.39	0.99, 1.97	0.061	2.30	1.79, 2.95	<0.001*
^‡^Model 2	1.11	0.78, 1.58	0.571	1.52	1.16, 2.00	0.003*
^§^Model 3	1.14	0.79, 1.64	0.472	1.56	1.19, 2.05	0.001*
High-teen NTG						
^†^Model 1	1.08	0.69, 1.67	0.748	1.55	1.11, 2.15	0.010*
^‡^Model 2	0.90	0.59, 1.42	0.639	1.20	0.85, 1.70	0.309
^§^Model 3	0.94	0.59, 1.51	0.800	1.18	0.84, 1.66	0.345

NTG, normal tension glaucoma. Actual hypertension: subjects with systolic/ diastolic blood pressure ≥ 140/90 mmHg without antihypertensive medication. Taking antihypertensive medication: subjects currently on medication.

*P < 0.05.

^†^Model 1: crude odd ratios.

^‡^Model 2: adjusted for age, and sex.

^§^Model 3: adjusted for age, sex, smoking status, refractive status, and education level.

## Discussion

The lamina cribrosa, a modified extension of the peripapillary scleral flange, forms a barrier between the intraocular space and retrobulbar space. Therefore, its ability to withstand the pressure difference between the two compartments and to maintain its shape is important in protecting the structures that pass through it, mainly the retinal ganglion axons [31, 32]. Recently, several population-based studies on the relationship between TLCPD and glaucoma have been conducted [33–37]. Moreover, an increasing body of research suggests that low CSFP and increased TLCPD level is an important risk factor for the development of open-angle glaucoma, especially in subjects with NTG [3–10]. Our study also showed that subjects with NTG had higher TLCPD than that of normal controls. However, when differentiated into low-teen and high-teen NTG groups, TLCPD was more strongly associated with the prevalence of glaucoma when compared with IOP in the high-teen IOP group but not the low-teen IOP group.

In the normal population, the weighted mean CSFP value was 11.69 ± 0.04 mmHg, which is similar to previously reported mean CSFP values measured directly by lumbar puncture [6–8]. Compared with previous population-based studies that used the same estimation formula, the weighted mean TLCPD value in the normal population (2.31 ± 0.06 mmHg), as well as the weighted mean CSFP value, was also very similar. Moreover, the ranges from minimum to maximum value for the CSFP and TLCPD were much greater (-1.80–23.91 mmHg and -14.61–19.11 mmHg, respectively) than that of IOP (6.00–21.00 mmHg). As reported previously [36, 37], such a relatively large range of values indicates that CSFP and TLCPD—and TLCPD to a slightly greater degree than CSFP—may contribute more to the development of glaucomatous optic neuropathy than to IOP, especially in patients with NTG. When the mean values of the three pressure parameters in this study were compared between normal controls and NTG patients, a greater TLCPD, higher IOP, and lower CSFP in NTG were noted. However, when NTG patients were divided into high-teen and low-teen IOP groups, high TLCPD was associated with the presence of glaucoma in the high-teen IOP group but not the low-teen IOP group.

Interestingly, a statistically significant association between presence of NTG and hypertension instead of TLCPD was found in the low-teen IOP group. Previously, Yamagami *et al*.[21] demonstrated that the influence of IOP on visual field defect was greater in NTG with high-teen IOP patients compared with low-teen IOP patients. Also, by demonstrating that retinal nerve fiber layer defect patterns differ between low-teen and high-teen NTG groups, Kim *et al*.[19] suggested that non-pressure factors, presumably hypertension, may contribute to the development of glaucomatous optic neuropathy in low-teen NTG patients. In more recent paper, simultaneous venous pulsation, which is thought to be closely related with the status of optic nerve circulation, was reported to be significantly decreased in the low-teen NTG subjects when compared to the normal and high-teen NTG patients [38]. While the association between vascular factors and low-teen OAG seems somewhat evident, relationship between CSFP and blood pressure, as reported to be positive in several studies, should also be taken into consideration. Recently, however, Fleischman et al.[39] demonstrated that CSFP did not differ between hypertensive and non-hypertensive patients, indicating that blood pressure level may not be relevant to CSFP. Overall, our results, together with those reported previously, strongly suggest that vascular factors are more closely associated with low-teen NTG compared with IOP and, and that TLCPD may not be an important pathogenetic factor. Conversely, increased TLCPD may be an important factor in high-teen NTG individuals.

Subgroup analysis from participants with hypertension revealed that taking antihypertensive medication were significantly associated with prevalence of low-teen NTG, while actual hypertension, BP exceeding 140/90 mmHg in the absence of antihypertensive medication, did not show significant association with prevalence of NTG. Previous studies argued on the exact pathogenetic role of hypertension on development of NTG, and some insisted the importance of habitual or episodic systemic hypotension that may lead to sudden drop of ocular perfusion pressure in optic nerve head [40, 41]. Such condition is much more likely to occur in patients taking antihypertensive medication than those who are not. While stiffening and atherosclerotic changes of retinal vessels may also contribute to the pathogenesis of NTG [42, 43], our findings implicate that susceptibility to optic disc hypoperfusion in patients taking antihypertensive medication may play a significant role in the development of glaucomatous optic neuropathy, especially in those with low-teen baseline IOP. However, much more complex underlying mechanism is yet to be defined on how hypertension contributes to the pathogenesis of NTG, and further prospective or longitudinal studies are needed to reveal the specific associations between different status of hypertension and NTG.

The strength of the present study is that it was based on a large population sample, minimizing selection bias. An additional strength is that any individuals previously diagnosed with glaucoma or who were on glaucoma medication were excluded from the study. Therefore, the subjects included in the two NTG groups were newly diagnosed, treatment-naïve patients with an initial IOP level in the low or high teens. However, this study also had several potential limitations. First, the CSFP parameter used in this study was calculated using a previously published formula. The pilot study in which the formula was developed included a relatively small number of subjects, and all participants were neurological patients [22]. However, previous large population-based studies have estimated the validity of this formula and revealed that the estimated CSFP was lower and TLCPD higher in subjects with glaucoma compared with the normal population [33–37], which is consistent with previous clinical studies. Considering the fact that the formula was developed based on a Chinese population and was validated in Chinese and Indian populations, its application to the Korean population may not cause problems related to ethnic differences. Although the positive association was revealed between estimated TLCPD and NTG in this study, TLCPD is a very complicated condition to define and to measure because of its dynamic nature. Several previous studies have investigated its relationship with OAG using CSFP obtained from lumbar puncture [6–9]. Meanwhile, Killer and his colleagues suggested the existence of orbital compartmentalization and insisted that CSFP around the optic nerve head differs from the lumbar puncture opening pressure [44–46]. Taking into account such poorly understood current concept on orbital CSFP and TLCPD, cautious interpretation is required when looking at the results of our study, since the estimated CSFP values derived from the formula, despite its validation from previous studies, may not exactly represent the true orbital CSFP in biomechanical models. Second, OAG with a normal IOP level in this study may not represent NTG precisely. Due to the limitations of the epidemiologic study design, it would have been difficult to define NTG in this study. One baseline IOP measurement may not be sufficient to represent a type of glaucoma. However, the relatively large number of subjects in this study would, at least in part, compensate for this limitation, and most of the OAG patients with a normal IOP level in this study would have NTG. Third, the findings reported herein are cross-sectional and observational. Although the study population was nationwide and sampled randomly, the cross-sectional nature of the study precludes the determination of a direct association between TLCPD and NTG. Future longitudinal studies should reveal the exact association.

In conclusion, TLCPD showed a stronger association with the prevalence of NTG compared with IOP in the high-teen IOP group. In the low-teen IOP group, however, neither IOP nor TLCPD showed a significant relationship with the prevalence of NTG. Instead, the presence of hypertension was significantly correlated with the prevalence of glaucoma. Our data support the hypothesis that different mechanisms may exist among NTG subjects, *i*.*e*., pressure-dependent factors in high-teen NTG and non-pressure-dependent factors in low-teen NTG patients. Future studies should aim to reveal the exact pathophysiology of development of optic nerve damage in low-teen and high-teen NTG patients.
